# Symphysis-fundus height measurement to predict small-for-gestational-age status at birth: a systematic review

**DOI:** 10.1186/s12884-015-0461-z

**Published:** 2015-02-10

**Authors:** Aase Serine D Pay, Johanna Wiik, Bjørn Backe, Bo Jacobsson, Annika Strandell, Atle Klovning

**Affiliations:** Department of Obstetrics and Department of Gynecology, Women’s and Children’s Division, Oslo University Hospital, Oslo, Norway; Department of International Public Health, Norwegian Institute of Public Health, Oslo, Norway; Department of Obstetrics and Gynecology, Institute of Clinical Sciences, Sahlgrenska Academy, Gothenburg, Sweden; Department of Laboratory Medicine, Children’s and Women’s Health, Norwegian University of Technology and Science, Trondheim University Hospital, Trondheim, Norway; Department of Genes and Environment, Norwegian Institute of Public Health, Oslo, Norway; Department of General Practice, Institute of Health and Society, Faculty of Medicine, University of Oslo, Oslo, Norway

**Keywords:** Small-for-gestational-age, Symphysis-fundus height, Pregnancy surveillance, Fetal growth, Fetal growth restriction

## Abstract

**Background:**

Fetal growth restriction is among the most common and complex problems in modern obstetrics. Symphysis-fundus (SF) height measurement is a non-invasive test that may help determine which women are at risk. This study is a systematic review of the literature on the accuracy of SF height measurement for the prediction of small-for-gestational-age (SGA) status at birth in unselected and low-risk pregnancies.

**Methods:**

The Medline, Embase, Cinahl, SweMed, and Cochrane Library databases were searched with no limitation on publication date (through September 2014), which returned 722 citations. Two reviewers then developed a short list of 51 publications of possible relevance and assessed them using the following inclusion criteria: cohort study of test accuracy performed in a routine prenatal care setting; SF height measurement for all participants; classification of SGA, defined as birth weight (BW) < 10th, 5th, or 3rd percentile or ≥ one or two standard deviations below the mean; study conducted in Northern, Western, or Central Europe; USA; Canada; Australia; or New Zealand; and sufficient data for 2 × 2 table construction. Quality of the included studies was assessed in duplicate using criteria suggested by the Cochrane Collaboration. Review Manager 5.3 software was used to analyze the data, including plotting of summary receiver operating curve spaces.

**Results:**

Eight studies were included in the final dataset and seven were included in summary analyses. The sensitivity of SF height measurement for SGA (BW < 10^th^ percentile) prediction ranged from 0.27 to 0.76 and specificity ranged from 0.79 to 0.92. Positive and negative likelihood ratios ranged from 1.91 to 9.09 and from 0.29 to 0.83, respectively.

**Conclusions:**

SF height can serve as a clinical indicator along with other clinical findings, information about medical conditions, and previous obstetric history. However, SF height has high false-negative rates for SGA. Clinicians must understand the limitations of this test.

The protocol has been registered in the international prospective register of systematic reviews, PROSPERO (Registration No. CRD42014008928, http://www.crd.york.ac.uk/prospero/display_record.asp?ID=CRD42014008928).

**Electronic supplementary material:**

The online version of this article (doi:10.1186/s12884-015-0461-z) contains supplementary material, which is available to authorized users.

## Background

Screening for fetal growth restriction (FGR) is one of the main purposes of antenatal care.

FGR is used to describe a fetus that did not reach its genetic growth potential and is associated with increased risks of morbidity and mortality, as well as adverse effects in childhood and later life [1-4]. Because no unanimously agreed-upon definition of FGR currently exists, small-for-gestational-age (SGA) is often used as a proxy. SGA is defined as weight below a specific percentile for gestational age, usually the 10th percentile. Although not all SGA neonates are pathologically growth restricted, detection of this group aims to facilitate the identification of at-risk pregnancies requiring further investigation due to potential FGR. Early identification and appropriate management of FGR can reduce perinatal morbidity and mortality [5].

In Scandinavia, screening relies on routine measurement of SF height, complemented by ultrasound measurement of fetal size in women with pregnancy complications or with a relevant history or clinical evidence of FGR [6-8]. SF height is a technique involving measurement of the maternal abdomen from the symphysis pubis to the uterine fundus with a tape measure. The measurement is plotted on a curve and compared with the distribution of the reference population [9,10]. If the recorded measurement is below acceptable limits according to the reference curves, further investigations of fetal growth and well-being are to be performed, including ultrasound estimations, uteroplacental and fetoplacental flow evaluations by Doppler, as well as cardiotocography.

Despite the routine use of SF height to predict SGA at birth, evidence for this method remains unclear. To date there is insufficient evidence from high quality trials to fully evaluate the effect of routine use of SF height during prenatal care on pregnancy outcomes [11]. Several studies have examined the accuracy of SF height in predicting SGA status at birth, but inconsistency in the results has been observed [12]. Most SF height research has been conducted in hospital-based settings and has investigated the relationship between SF height and SGA status in high risk populations [13-15]. Because of a different prevalence (pre-test probability) of SGA, results from hospital-based studies cannot be extrapolated to primary care settings.

### Objectives

In this systematic review we aim to assess the sensitivity and specificity of SF height for the prediction of SGA status at birth in unselected and low-risk pregnancies.

## Methods

### Criteria for considering studies for this review

Studies were selected for inclusion in the review according to the population, index test, target condition, reference standard, outcome measure, and study design.

#### Population

Studies examining singleton pregnancies in unselected or low-risk populations, conducted in comparable health care systems to Scandinavia (Northern, Western and Central Europe, USA, Canada, Australia, and New Zealand).

#### Index test

SF measurement compared to the SF distribution of the population.

#### Target condition

SGA or FGR.

#### Reference standard

Diagnosis of FGR or SGA, defined as birth weight (BW) < 10th, 5th, or 3rd percentile, or ≥ one or two standard deviations (SDs) below the mean (performed postnatally).

#### Outcome measures

Data required to populate 2 × 2 contingency tables.

#### Study design

Diagnostic cohort studies.

### Search methods for identification of studies

Electronic databases (PubMed, Medline, Embase, CINAHL, Cochrane Library, and SweMEd) were searched to identify eligible diagnostic studies from the earliest year possible through September 2014. The search strategy was developed for PubMed and modified for use in other databases (see Additional file 1). The reference lists of all included publications and relevant systematic reviews were checked and forward citation searches were performed.

#### Electronic searches

The search strategy involved combinations of SF-related terms appearing in subject headings and as keywords. Our Medline search query was (fund* adj height*) OR (symph* adj fund*) OR (uter* adj height*) OR (symph* adj height*) OR (gravidogram*) OR (uterus fundus height*) OR (uter* fund* height*). We conducted our search and reported our findings according to the Meta-Analysis of Observational Studies in Epidemiology and Preferred Reporting Items for Systematic Reviews and Meta-Analyses statements [16-18].

### Data collection and analysis

#### Study selection

A list of articles meeting the inclusion criteria based on abstracts was compiled. The full texts of these studies and those of uncertain relevance were retrieved. Two reviewers (ASDP and JW) independently evaluated the studies’ fulfillment of the inclusion criteria, with any discrepancy discussed with a third reviewer until a final set of relevant studies was agreed upon.

#### Data extraction and management

The following data were extracted from all selected studies: general information (first author, publication year, country of investigation), population (health care setting, number of participants, level of risk), study design (design, data collection), characteristics of SF height test (SF height curve, cut-off points), reference standard (SGA definition) and results (data required for the construction of 2 × 2 contingency tables). Data were entered into a database using Review Manager 5.3 software.

#### Assessment of methodological quality

The quality of each included study was assessed by two review authors (ASDP, JW) using the QUality Assessment of Diagnostic Accuracy Studies (QUADAS-2) checklist [19,20]. The QUADAS-2 checklist asks signaling questions in four risks of bias domains relating to patient selection, index test, reference standard, and flow and timing. Each domain is assessed in terms of risk of bias, and the first three domains are also assessed in terms of applicability. The review authors classified each item as “yes” (adequately addressed), “no” (inadequately addressed), or “unclear” (inadequate detail presented to allow a judgment to be made). The QUADAS-2 tool is shown in Additional file 2.

#### Statistical analysis and data synthesis

Data on sensitivity, specificity, and true-positive, false-positive, true-negative, and false-negative results were taken directly from the source papers or, if necessary, calculated from the data provided. Positive likelihood ratios (PLRs), negative likelihood ratios (NLRs), diagnostic odds ratios (DORs), and 95% confidence intervals (CIs) were calculated.

An LR describes how many times more likely it is that a person with the target condition will receive a particular test result than will a person without it. Categorization of LRs was adopted from Deeks et al. [21] where PLRs > 10 or NLRs < 0.1 are considered to provide convincing diagnostic evidence. The DOR is commonly used as an overall indicator of diagnostic performance and calculated as the odds of a positive test result among those with the target condition, divided by the odds of a positive test result among those without the condition. As a general rule, a DORs > 100 indicates high accuracy, values of 25–100 indicate moderate accuracy, and those < 25 indicates that the test is not useful [21].

The data were displayed graphically on forest and summary receiver operating characteristic (SROC) plots [22]. The SROC curve was fitted using the hierarchical bivariate random-effects method [23]. For studies that used more than one SF threshold, the analysis was based on the cut-off point of “one value < 10^th^ percentile”.

#### Investigation of heterogeneity

Both clinical and statistical heterogeneity were evaluated. Assessment of clinical heterogeneity involved comparison of SF reference curves, cut-off criteria used to identify abnormal results, and SGA definitions. Assessment of statistical heterogeneity involved visual inspection of forest plots and calculation of the inconsistency index (I^2^), which describes the percentage of total variation across studies that is due to heterogeneity, rather than chance [24].

## Results

**Figure 1:**
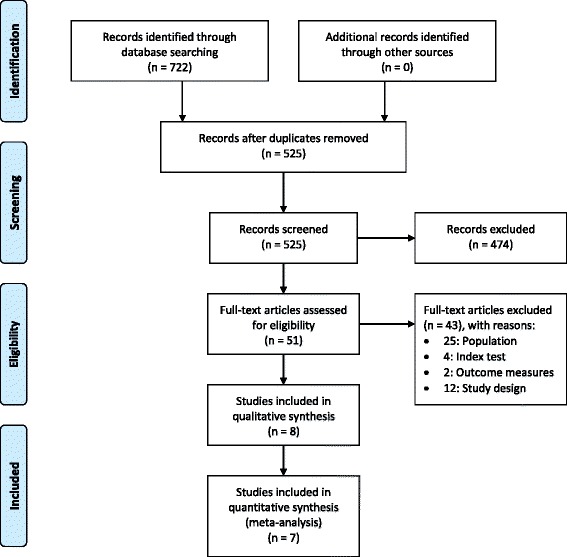
**Flow diagram.** PRISMA flow diagram of studies through the review.

Initial database searches retrieved 722 citations of which 525 citations remained after duplicates were removed (Figure 1). Screening of the titles and abstracts identified 51 potentially relevant articles that were retrieved in full text format. Forward and backward citation tracking did not result in the identification of additional relevant articles. Eight articles were included in final analyses. Additional file 3 lists the reasons for excluding 43 articles on the basis of study population, design or outcome measures.

### Included studies

Characteristics of included studies [25-32] are presented in Table 1. All studies were published before 1991. Most studies used locally derived SF curves. Different cut-off criteria were used to identify abnormal results, including one value < 10th percentile; two consecutive or three isolated values < 10th percentile; one value > 2 cm below the mean; one value > 2 cm below the mean or three static or falling values; and one value > two SDs below the mean. Definitions of SGA included BW < 10th percentile, < 5th percentile, and ≥ two SDs below the mean, according to local standards.

**Table 1:** **Characteristics of included studies**

**Study**	**Year**	**Country**	**n**	**SF curve**	**Cut-off defining abnormal test**	**Definition of SGA**
Calvert et al. [25]	1982	Great Britain	381	local	One value < 10th percentile, two consecutive values or three isolated values < 10th percentile, one value ≥ 3 cm below mean or three consecutive static or falling values	< 10th percentile, < 5th percentile
Cnattingius et al. [26]	1988	Sweden	3038	Westin	One or more values ≥ 3 cm below mean or falling or static values	≥ two SDs below mean
Jensen et al. [27]	1991	Norway	831	Westin	One or more values ≥ 3 cm below mean	< 10th percentile
Pearce et al. [28]	1987	Great Britain	699	local	One value < 10th percentile	< 10th percentile
Persson et al. [29]	1986	Sweden	2919	local	One value > two SDs below mean	< 10th percentile
Rogers et al. [30]	1985	Great Britain	250	local	One or more values ≥ 3 cm below mean or three consecutive static or falling values	< 10th percentile
Rosenberg et al. [31]	1982	Great Britain	761	local	Two consecutive or three isolated values < 10th percentile	< 10th percentile
Stuart et al. [32]	1989	Great Britain	1139	Calvert	One or more values < 10th percentile after 26 weeks or falling or static values	< 10th percentile

n, number of patients; SD, standard deviation; SF, symphysis-fundus; SGA, small-for-gestational-age.

### Methodological quality of included studies

The QUADAS-2 ratings of risk of bias and study applicability are shown in Table 2. Based on the inclusion criteria, no included study had a case–control design. All studies avoided inappropriate exclusions. Six of the eight studies used consecutive or random recruitment of participants. The two remaining studies [30,32] did not report such information and were considered to be at unclear risk of patient selection bias. Most studies had a low risk of bias due to patient flow and timing; seven of eight studies involved the analysis of all recruited participants and one analysis included 78% of recruited participants [32]. Studies included in this review had a low risk of bias for the conduct of the reference standard. All studies used pre-specified index test thresholds. No study reported blinding to test results, but BW is objective and should not result in bias. Regarding the applicability of studies to the review questions, no study raised concern about the index test, reference standard or patient selection.

**Table 2:** **Risk of bias and applicability concerns summary**

**Study**	**Risk of bias**				**Applicability concerns**		
	**Patient selection**	**Index test**	**Reference standard**	**Flow and timing**	**Patient selection**	**Index test**	**Reference standard**
Calvert et al. [25]	low	low	low	low	low	low	low
Cnattingius et al. [26]	low	low	low	low	low	low	low
Jensen et al. [27]	low	low	low	low	low	low	low
Pearce et al. [28]	low	low	low	low	low	low	low
Persson et al. [29]	low	low	low	low	low	low	low
Rogers et al. [30]	unclear	low	low	low	low	low	low
Rosenberg et al. [31]	low	low	low	low	low	low	low
Stuart et al. [32]	unclear	low	low	unclear	low	low	low

Risk of bias and applicability concerns summary based on the QUADAS-2 checklist.

### Statistical analysis

Tables 3, 4, 5 display core information collected from all included studies according to the SGA definition used by the study authors.

**Table 3:** **Accuracy of symphysis-fundus height in predicting small-for-gestational-age status (birth weight < 10th percentile) with 95% confidence intervals**

**SF cut-off**	**n**	**Sensitivity**	**Specificity**	**Positive LR**	**Negative LR**	**DOR**
**One value < 10th percentile**						
Calvert et al. [25]	381	0.64 (0.49-0.78)	0.79 (0.74-0.83)	3.05 (2.26-4.12)	0.45 (0.30-0.67)	6.76 (3.48-13.14)
Pearce et al. [28]	699	0.76 (0.66-0.84)	0.79 (0.75-0.82)	3.61 (2.99-4.37)	0.30 (0.21-0.43)	11.89 (7.22-19.58)
Stuart et al. [32]	1139	0.51 (0.40-0.61)	0.88 (0.86-0.90)	4.19 (3.22-5.46)	0.56 (0.45-0.70)	7.45 (4.71-11.80)
**Two consecutive values or three isolated values < 10th percentile**						
Calvert et al. [25]	381	0.36 (0.22-0.51)	0.94 (0.91-0.96)	5.69 (3.21-10.07)	0.69 (0.55-0.86)	8.28 (3.90-17.58)
Rosenberg et al. [31]	761	0.56 (0.41-0.70)	0.85 (0.82-0.87)	3.65 (2.70-4.92)	0.52 (0.38-0.71)	7.01 (3.87-12.71)
**One value ≥ 3 cm below mean**						
Jensen et al. [27]	831	0.41 (0.31-0.51)	0.87 (0.85-0.90)	3.25 (2.38-4.42)	0.68 (0.57-0.80)	4.78 (3.01-7.59)
**One value ≥ 3 cm below mean or three consecutive static or falling values**						
Calvert et al. [25]	381	0.76 (0.60-0.87)	0.60 (0.55-0.66)	1.91 (1.54-2.36)	0.40 (0.24-0.68)	4.72 (2.31-9.64)
Rogers et al. [30]	250	0.73 (0.52-0.88)	0.92 (0.88-0.95)	9.09 (5.51-15.00)	0.29 (0.16-0.55)	31.06 (11.53-83.72)
**One value > two SDs below mean**						
Persson et al. [29]	2919	0.27 (0.21-0.32)	0.88 (0.87-0.89)	2.22 (1.77-2.78)	0.83 (0.77-0.90)	2.66 (1.97-3.58)

DOR, diagnostic odds ratio; LR, likelihood ratio; n, number of patients; SD, standard deviation; SF, symphysis-fundus.

Accuracy of symphysis-fundus height in predicting small-for-gestational-age status (birth weight < 10th percentile) with 95% confidence intervals.

**Table 4:** **Accuracy of symphysis-fundus height in predicting small-for-gestational-age status (birth weight < 5th percentile) with 95% confidence intervals**

**SF cut-off**	**n**	**Sensitivity**	**Specificity**	**Positive LR**	**Negative LR**	**DOR**
**One value < 10th percentile**						
Calvert et al. [25]	381	0.60 (0.39-0.79)	0.76 (0.71-0.80)	2.51 (1.74-3.64)	0.53 (0.32-0.85)	4.78 (2.07-11.04)
**Two consecutive values or three isolated values < 10th percentile**						
Calvert et al. [25]	381	0.36 (0.18-0.57)	0.92 (0.89-0.95)	4.58 (2.43-8.61)	0.69 (0.52-0.93)	6.59 (2.67-16.26)
**One value ≥ 3 cm below mean or three consecutive static or falling values**						
Calvert et al. [25]	381	0.72 (0.51-0.88)	0.58 (0.53-0.63)	1.72 (1.31-2.26)	0.48 (0.26-0.91)	3.57 (1.46-8.77)

DOR, diagnostic odds ratio; LR, likelihood ratio; n, number of patients; SD, standard deviation; SF, symphysis-fundus.

Accuracy of symphysis-fundus height in predicting small-for-gestational-age status (birth weight < 5th percentile) with 95% confidence intervals.

**Table 5:** **Accuracy of symphysis-fundus height in predicting severe small-for-gestational-age status (birth weight ≥ two standard deviations below the mean) with 95% confidence intervals**

**SF cut-off**	**n**	**Sensitivity**	**Specificity**	**Positive LR**	**Negative LR**	**DOR**
**One value ≥ 3 cm below mean or falling or static values**						
Cnattingius et al. [26]	3038	0.59 (0.39-0.78)	0.97 (0.96-0.98)	19.83 (13.65-28.79)	0.42 (0.27-0.66)	47.21 (21.30-104.62)

DOR, diagnostic odds ratio; LR, likelihood ratio; n, number of patients; SD, standard deviation; SF, symphysis-fundus.

Accuracy of symphysis-fundus height in predicting severe small-for-gestational-age status (birth weight ≥ two standard deviations below the mean) with 95% confidence intervals.

#### Accuracy of SF height for the prediction of SGA defined as BW < 10th percentile

**Figure 2:**
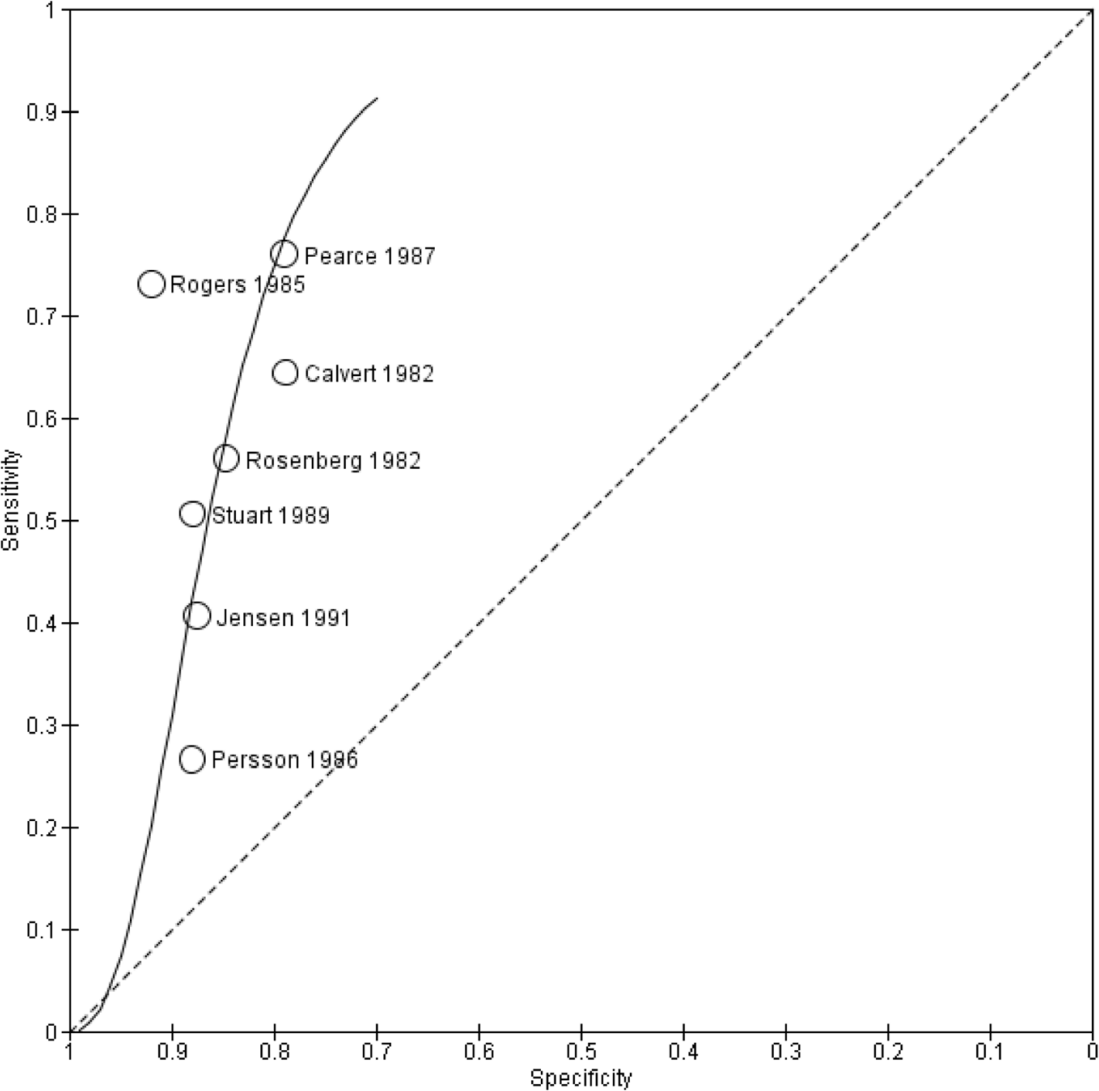
**Summary receiving operating characteristic plot.** Summary receiving operating characteristic plot of symphysis-fundus height measurement for the prediction of small-for-gestational-age status (birth weight < 10th percentile).

Seven studies assessed the accuracy of SF height for the prediction of SGA defined as BW < 10th percentile. Sensitivities ranged from 0.27 to 0.76 and specificities ranged from 0.79 to 0.92. All studies produced DORs exceeding 1 and CIs that did not include 1, implying that the positive association of SF height with SGA was not due to chance alone. PLRs exceeded 1 in all studies, indicating that abnormal SF height values were associated with SGA status at birth. However all PLRs were <10, the threshold generally accepted for a useful test. The same seven studies reported NLRs < 1, indicating that normal SF height values were correctly associated with the absence of SGA. However, no study met the accepted criterion of NLR < 0.1 in this group of women. The SROC curve (Figure 2) constructed using data from these studies lies to the left of the diagonal, signifying that the SF height test has value. The I^2^ value was typically high (98%). Given the small number of included studies (and thus low statistical power), subgroup analyses and covariate hierarchical modeling to investigate heterogeneity were not performed.

#### Accuracy of SF height for the prediction of SGA defined as BW <5th percentile

One study assessed the accuracy of SF height for the prediction of SGA defined as BW < 5th percentile. This study used several cut-off points, with stricter criteria yielding lower sensitivity and higher specificity values. NLRs and PLRs did not meet the accepted criteria for classification of SF height measurement as a useful test.

#### Accuracy of SF height for the prediction of SGA defined as BW ≥ 2 SDs below the mean

One study assessed the outcome of SGA defined as BW ≥ 2 SDs below the mean. For a less strict SF cut-off point (one value > 2 cm below mean or falling or static values), the authors reported low sensitivity (59%) and high specificity (97%). The PLR exceeded 10, but the NLR did not meet the required criterion of <0.1.

## Discussion

SF height measurement seems to have some significance for the prediction of SGA defined as BW < 10th percentile. All studies reported DORs > 1. The SROC curve (Figure 2) lies to the left of the diagonal, signifying that the SF height test has value. Adequate levels of sensitivity appear to be achieved at the expense of lower specificity, with higher numbers of false-positive SF results. The study of Rogers et al. [30] positioned at the upper left of the SROC curve produced the most significant results supporting the use of SF height. Its false negative rate of only seven is likely to be due to the small size of the study. In contrast, the study of Persson et al. [29] is the largest study and has the narrowest CI. Its sensitivity and specificity lies along the SROC line, adding weight to our findings.

For the prediction of SGA defined as BW < 5th percentile and BW ≥ 2 SDs below the mean, no summary measure could be performed due to the insufficient number of studies assessing these outcomes. Further assessment of the predictive value of SF in prediction of SGA defined as BW < 5th percentile and BW ≥ 2 SDs below the mean is required.

The diagnostic accuracy of SF height in other populations of pregnant women has recently been reviewed. Goto [33] assessed the diagnostic value of SF height, mainly in developing countries. However, this review included studies across a wide range of ethnic groups, clinical settings and disease spectrums. Despite such a diverse case mix, the study did not assess its effect on the pooled estimates, thus making it difficult to interpret its finding in a low-risk setting. In view of these limitations, we applied more strict inclusion criteria in our study, focusing mainly on a more homogenous and relevant population.

### Strengths and weaknesses of the review

The majority of studies available in this systematic review were conducted in the 1980s. Given the limited amount of data available for the accuracy of SF height measurement, we did not discard studies based solely on year of publication. All included studies had low concern regarding applicability, implying that evidence is relevant to current practice. The focus on nations with comparable health systems means that the findings may not be relevant to different and less well-resourced national health systems.

Many parameters involving the performance of SF height measurement, such as technique, frequency of measurement, and performer’s experience, potentially affect test accuracy. Unfortunately, we did not have detailed information about the test conditions, limiting our ability to explore the effects of potential differences in methods. As no universal SGA definition has been established, the studies included in this review may also have been biased by the choice of reference test. Our inclusion criteria required postnatal confirmation of SGA classification. All studies fulfilled this requirement, but most did not provide information about how gestational age was determined or which BW reference were used to classify SGA status postnatally.

This review focused on the role of SF height in detecting SGA as a proxy for FGR. However, FGR can exist without SGA. The role of SF height in this setting remains undefined because all SF height studies in this review used SGA as an outcome. Customized SF charts (adjusted for ethnicity, parity, and body mass index) are said to be better predictors of FGR [34]. Furthermore, this review did not address the issue of effect, for which additional studies would be needed to assess the role of SF height.

Ultimately, the lack of large cohort studies conducted in routine prenatal care setting that were suitable for our analysis was the main limitation of this review.

### Applicability of findings to clinical practice and policy

SF height can be the first parameter raising suspicion of FGR. We have previously discussed the limitations of the study populations. However, our results can be applied to low-risk and unselected pregnancies in routine prenatal care setting, which is useful for general practitioners and midwifes to assure the identification of pregnancies at risk of SGA.

We found that the SF height test had a sensitivity ranging from 0.27 to 0.76, which means it potentially fails to identify over 70% of pregnancies affected by SGA. This is important to consider in counselling of pregnant women. However, in clinical practice the SF height test is not carried out in isolation and the combination of other clinical findings, medical conditions and previous obstetric history, together will contribute to estimating the likelihood of being at risk for SGA.

Our results show that the SF height test has a high degree of specificity (≥80% in all studies), indicating that few pregnancies not characterized by SGA are referred for ultrasound examination in practice. However, in this case over-referral or the misidentification of pregnancies as at risk is of less concern than the failure to identify pregnancies at risk.

Primary screening should emphasize the importance of sensitivity over specificity to identify almost all at-risk participants. No test is perfect and there will always be problems with incorrect results, e.g., anxiety and unnecessary intervention due to a false-positive result or a false sense of security caused by a false-negative result. A positive SF screening result can usually be confirmed or refuted with further evaluation of fetal growth and well-being by a specialist.

## Conclusion

### Implications for practice

SF height can play a role in clinical practice. It is a non-invasive, simple, and inexpensive method. However, it has low sensitivity. Other techniques that could improve upon this limitation (e.g., routine ultrasound in the third trimester) have not been implemented in the routine prenatal care setting [35]. We recommend the continued use of SF height measurement in clinical practice as one of several indicators for referral to an obstetric care unit. However, clinicians must understand the limitations of the test.

### Implications for research

Further studies including larger numbers of patients and better standardized reporting criteria are desirable. The accuracy of adjusted over unadjusted SF curves needs to be evaluated.

## Additional files

Additional file 1:
**Search strategy for electronic databases.**
Additional file 2:
**QUADAS-2 tool.**
Additional file 3:
**Articles excluded on the basis of the full-text from this study and reason for exclusion.**

## Abbreviations

BW: Birth weight
CI: Confidence interval
DOR: Diagnostic odds ratio
FGR: Fetal growth restriction
NLR: Negative likelihood ratio
PLR: Positive likelihood ratio
PRISMA: Preferred reporting items for systematic reviews and meta-analyses
QUADAS: Quality assessment of diagnostic accuracy studies
SD: Standard deviation
SF: Symphysis-fundus
SGA: Small-for-gestational-age
SROC: Summary receiver operating characteristic
