# Supplementary material for: Symphysis-fundus height measurement to predict small-for-gestational-age status at birth: a systematic review
Source: BMC Pregnancy Childbirth. 2015 Feb 10;15:22. doi: 10.1186/s12884-015-0461-z (PMC4328041; doi:10.1186/s12884-015-0461-z)
Supplement: Additional file 1: — Search strategy for electronic databases. [file 12884_2015_461_MOESM1_ESM.pdf]

Medline in-process and other non-indexed citations, Ovid Medline (R) (1946-), and Embase (1974-)

1 (fund* adj height*).mp.

2 (symph* adj fund*).mp.

3 (uter* adj height*).mp.

4 (symph* adj height*).mp.

5 (gravidogram).mp.

6 1 or 2 or 3 or 4 or 5

Cinahl (1981-)

S1 (MH "Fundal Height")

S2 fund* height*

S3 symph* fund*

S4 uter* height*

S5 symph* height*

S6 gravidogram

S7 S1 or S2 or S3 or S4 or S5 or S6

Swemed+ (1977-)

1 exp:”fundal height”

2 fund* height*

3 symph* fund*

4 uter* height*

5 symph* height*

6 symfys* fundus*

7 symfys*

8 gravidogram

9 1 or 2 or 3 or 4 or 5 or 6 or 7 or 8

The Cochrane Library

(fund* NEXT height*):ti,ab,kw or (symph* NEXT fund*):ti,ab,kw or (uter* NEXT height*):ti,ab,kw or (symph* NEXT height*):ti,ab,kw or (gravidogram).ti,ab,kw
