# Supplementary material for: Symphysis-fundus height measurement to predict small-for-gestational-age status at birth: a systematic review
Source: BMC Pregnancy Childbirth. 2015 Feb 10;15:22. doi: 10.1186/s12884-015-0461-z (PMC4328041; doi:10.1186/s12884-015-0461-z)
Supplement: Additional file 2: — QUADAS-2 tool. [file 12884_2015_461_MOESM2_ESM.pdf]

Domain	Signaling Questions*	Risk of Bias†	Applicability Concerns†
Patient Selection	(1) Was a consecutive or random sample of patients enrolled?	Could the selection of patients have introduced bias?	Are there concerns that the included patients and setting do not match the review question?
	(2) Was a case-control design avoided?		
	(3) Did the study avoid inappropriate exclusions?		
Index Test	(1) Were the index test results interpreted without knowledge of the results of the reference standard?	Could the conduct or interpretation of the index test have introduced bias?	Are there concerns that the index test, its conduct, or interpretation differ from the review question?
	(2) If a threshold was used, was it pre-specified?		
Reference Standard	(1) Is the reference standard likely to correctly classify the target condition?	Could the reference standard, its conduct, or its interpretation have introduced bias?	Are there concerns that the target condition as defined by the reference standard does not match the question?
	(2) Were the reference standard results interpreted without knowledge of the results of the index test?		
Flow and Timing	(1) Was there an appropriate interval between index test and reference standard?	Could the patient flow have introduced bias?	Not applicable
	(2) Did all patients receive a reference standard?		
	(3) Did all patients receive the same reference standard?		
	(4) Were all patients included in the analysis?		

* Signaling questions are rated “yes”, “no”, or “unclear”. † Risk of bias judgments and concerns related to applicability are rated “high”, “low” or “unclear”.

Reference: Whiting PF, Rutjes AW, Westwood ME, Mallett S, Deeks JJ, Reitsma JB, Leeflang MM, Sterne JA, Bossuyt PM: QUADAS-2: a revised tool for the quality assessment of diagnostic accuracy studies. Ann Intern Med 2011, 155(8):529-536.
