# Supplementary material for: Symphysis-fundus height measurement to predict small-for-gestational-age status at birth: a systematic review
Source: BMC Pregnancy Childbirth. 2015 Feb 10;15:22. doi: 10.1186/s12884-015-0461-z (PMC4328041; doi:10.1186/s12884-015-0461-z)
Supplement: Additional file 3: — Articles excluded on the basis of the full-text from this study and reason for exclusion. [file 12884_2015_461_MOESM3_ESM.pdf]

Nr.	Study	Reason for exclusions
1.	Symphysis-fundal height measurement. The practising midwife 2000, 3(1):10.	Not an original study
2.	Adjahoto EO, Djossou KP, Hodonou KA: Prediction of fetal hypotrophy by uterine height. [French] Prediction de l'hypotrophie foetale par la hauteur uterine. J Gynecol Obstet Biol Reprod (Paris) 1999, 28(4):352-357.	Population not relevant
3.	Azziz R, Smith S, Fabro S: The development and use of a standard symphysial-fundal height growth curve in the prediction of small for gestational age neonates. International Journal of Gynecology and Obstetrics 1988, 26(1):81-87.	Study design not relevant
4.	Belizan JM, Villar J, Nardin JC: Diagnosis of intrauterine growth retardation by a simple clinical method: Measurement of uterine height. Am J Obstet Gynecol 1978, 131(6):643-646.	Population not relevant
5.	Berry M, Coyaji KJ, Gogoi MP, Kodkany BS, Parikh KS, Patel D, Rawal MY, Sengupta PC, Walvekar V, Dhillon BS: Foetal growth parameters--clinical versus ultrasonographic. Indian J Pediatr 1992, 59(1):91-101.	Index test not relevant
6.	Cnattingius S, Axelsson O, Eklund G, Lindmark G: Screening for intrauterine growth retardation in late pregnancy. Early Hum Dev 1985, 10(3-4):225-235.	Population not relevant
7.	Cox G, Walsh P, Stack J, Murphy H: The value of fundal height measurement in prediction of fetal growth retardation. Ir Med J 1983, 76(2):95-96.	Population not relevant
8.	Cronje HS, Bam RH, Muir A: Validity of symphysis fundus growth measurements. International Journal of Gynecology and Obstetrics 1993, 43(2):157-161.	Population not relevant
9.	Engstrom JL, Work Jr BA: Prenatal prediction of small- and large-for-gestational age neonates. Journal of obstetric, gynecologic, and neonatal nursing : JOGNN / NAACOG 1992, 21(6):486-495.	Population not relevant
10.	Feiks A, Gring H, Gruber W: The value of the symphysis-fundus measurement in prenatal care. [German] Stellenwert der Symphysen-Fundusmessung in der Schwangerenvorsorge. Gynakol Rundsch 1988, 28 Suppl 2:135-137.	Study design not relevant
11.	Fescina RH, Quevedo C, Martell M, Nieto F, Schwarcz R: Uterine height as a method of predicting fetal growth. [Spanish] Altura uterina como metodo para predecir el crecimiento fetal. Bol Oficina Sanit Panam 1984, Pan American Sanitary Bureau. 96(5):377-386.	Population not relevant
12.	Foley ME: Measurement of fundal height. Ir Med J 1991, 84(4):114.	Not an original study
13.	Gardosi J, Francis A: Controlled trial of fundal height measurement plotted on customised antenatal growth charts. Br J Obstet Gynaecol 1999, 106(4):309-317.	Study design not relevant
14.	Ghate M, Pratinidhi A, Gupte A: Risk prediction charts for low birth weight. Indian Pediatr 1996, 33(1):15-18.	Population not relevant

15.	Harding K, Evans S, Newnham J: Screening for the small fetus: A study of the relative efficacies of ultrasound biometry and symphysiofundal height. Aust N Z J Obstet Gynaecol 1995, 35(2):160-164.	Relevant data was not extractable
16.	Indraccolo U, Chiocci L, Rosenberg P, Nappi L, Greco P: Usefulness of symphysis-fundal height in predicting fetal weight in healthy term pregnant women. Clin Exp Obstet Gynecol 2008, 35(3):205-207.	Population not relevant
17.	Isager-Sally L, Nielsen PV, Andersen J: Symphysis/fundus measurements. Investigation of the method. [Danish]. Ugeskr Laeger 1985, 147(7):609-611.	Study design not relevant
18.	Kennedy I: The implications of introducing the symphyseal-fundal height-measurement. A prospective randomized controlled trial [2]. Br J Obstet Gynaecol 1992, 99(7):625-626.	Population not relevant
19.	Linasmita V: Antenatal screening of small-for-gestational age infants by symphysial-fundal height measurement. Journal of the Medical Association of Thailand = Chotmaihet thangphaet 1985, 68(11):587-591.	Population not relevant
20.	Linasmita V: Serial symphysis--fundal height measurements in detection of abnormal fetal growth. Journal of the Medical Association of Thailand = Chotmaihet thangphaet 1986, 69(11):585-589.	Population not relevant
21.	Linasmita V, Sugkraroek P: Normal uterine growth curve by measurement of symphysial-fundal height in pregnant women seen at Ramathibodi Hospital. Journal of the Medical Association of Thailand = Chotmaihet thangphaet 1984, 67 Suppl 2:22-26.	Population not relevant
22.	Lindhard A: The measurement of symphyseal-fundal height. Nurs Times 1990, 86(42):58-59.	Not an original study
23.	Lindhard A, Nielsen PV, Mouritsen LA, Zachariassen A, Sorensen HU, Roseno H: The implications of introducing the symphyseal-fundal height-measurement. A prospective randomized controlled trial. Br J Obstet Gynaecol 1990, 97(8):675-680.	Study design not relevant
24.	Mathai M, Jairaj P, Muthurathnam S: Screening for light-for-gestational age infants: A comparison of three simple measurements. Br J Obstet Gynaecol 1987, 94(3):217-221.	Population not relevant
25.	Mattioli KP, Sanderson M, Chauhan SP: Inadequate identification of small-for-gestational-age fetuses at an urban teaching hospital. International Journal of Gynecology and Obstetrics 2010, 109(2):140-143.	Population not relevant
26.	Mongelli M, Gardosi J: Estimation of fetal weight by symphysis-fundus height measurement. International Journal of Gynecology and Obstetrics 2004, 85(1):50-51.	Study design not relevant
27.	Norton R: The prediction of intrauterine growth retardation in remote area Aboriginal women using serial fundal-symphysial height measurements. Aust N Z J Obstet Gynaecol 1989, 29(3 II):306-307.	Population not relevant
28.	Ohwada M, Igarashi M: [Evaluation of measurement of maternal symphysis-fundus length as fetal growth screening method]. Nippon Sanka Fujinka Gakkai Zasshi 1983, 35(5):637-644.	Population not relevant
29.	Okonofua FE, Ayangade SO, Chan RCW, O'Brien PMS: A prospective comparison of clinical and ultrasonic methods of predicting normal and abnormal fetal growth. International Journal of Gynecology and Obstetrics 1986, 24(6):447-451.	Population not relevant

30.	Pattinson RC, Theron GB: Inter-observer variation in symphysis-fundus measurements: A plea for individualised antenatal care. S Afr Med J 1989, 76(11):621-622.	Population not relevant
31.	Pillay P, Janaki S, Manjila C: A comparative study of gravidogram and ultrasound in detection of IUGR. Journal of Obstetrics and Gynecology of India 2012, 62(4):409-412.	Population not relevant
32.	Pommier M, Escobedo F, Lowenberg E: Measurement of height of the uterine fundus for detection of retarded intrauterine growth. [Spanish] Estudio De La Altura Del Fondo Uterino Para La Deteccion Del Crecimiento Intrauterino Retardado. Ginecol Obstet Mex 1979, 46(276):253-259.	Population not relevant
33.	Reynolds JL, Kettner A, Burnett M, Cheang M: Can fundal height predict birth weight or twins? Can Fam Physician 1986, 32:55-60.	Unclear index test
34.	Roex A, Nikpoor P, Van Eerd E, Hodyl N, Dekker G: Serial plotting on customised fundal height charts results in doubling of the antenatal detection of small for gestational age fetuses in nulliparous women. Aust N Z J Obstet Gynaecol 2012, 52(1):78-82.	Study design not relevant
35.	Rondo PHC, Maia Filho NL, Valverde KK: Symphysis-fundal height and size at birth. International Journal of Gynecology and Obstetrics 2003, 81(1):53-54.	Population not relevant
36.	Rosendahl H, Kivinen S: Detection of small for gestational age fetuses by the combination of clinical risk factors and ultrasonography. European Journal of Obstetrics Gynecology and Reproductive Biology 1991, 39(1):7-11.	Index test not relevant
37.	Rosser J: Cochrane made simple. Symphysis-fundal height measurement. Practising Midwife 2000, 3(1):10-10.	Not an original study
38.	Secher NJ, Lundbye-Christensen S, Qvist I, Bagger P: An evaluation of clinical estimation of fetal weight and symphysis fundal distance for detection of SGA infants. European Journal of Obstetrics Gynecology and Reproductive Biology 1991, 38(2):91-96.	Population not relevant
39.	Theron GB, Theron AM, Odendaal HJ: Symphysis-fundus growth measurement followed by umbilical artery Doppler velocimetry to screen for placental insufficiency. International Journal of Gynecology and Obstetrics 2002, 79(3):263-264.	Population not relevant
40.	Tjon Ten AWE, Kusin JA, De With C: Fundal height measurement as an antenatal screening method. J Trop Pediatr 1985, 31(5):249-252.	Population not relevant
41.	Westin B: Gravidogram and fetal growth. Comparison with biochemical supervision. Acta Obstet Gynecol Scand 1977, 56(4):273-282.	Relevant data was not extractable
42.	Wise D, Engstrom JL: The predictive validity of fundal height curves in the identification of small- and large-for-gestational-age infants. Journal of obstetric, gynecologic, and neonatal nursing : JOGNN / NAACOG 1985, 14(2):87-92.	Study design not relevant
43.	Wright J, Morse K, Kady S, Francis A: Audit of fundal height measurement plotted on customised growth charts. MIDIRS Midwifery Digest 2006, 16(3):341-345.	Index test not relevant
